# Platelet Specific Promoters Are Insufficient to Express Protease Activated Receptor 1 (PAR1) Transgene in Mouse Platelets

**DOI:** 10.1371/journal.pone.0097724

**Published:** 2014-05-15

**Authors:** Amal Arachiche, María de la Fuente, Marvin T. Nieman

**Affiliations:** Department of Pharmacology, Case Western Reserve University, Cleveland, Ohio, United States of America; Emory University/Georgia Insititute of Technology, United States of America

## Abstract

The *in vivo* study of protease activated receptors (PARs) in platelets is complicated due to species specific expression profiles. Human platelets express PAR1 and PAR4 whereas mouse platelets express PAR3 and PAR4. Further, PAR subtypes interact with one another to influence activation and signaling. The goal of the current study was to generate mice expressing PAR1 on their platelets using transgenic approaches to mimic PAR expression found in human platelets. This system would allow us to examine specific signaling from PAR1 and the PAR1-PAR4 heterodimer *in vivo*. Our first approach used the mouse GPIbα promoter to drive expression of mouse PAR1 in platelets (GPIbα-Tg-mPAR1). We obtained the expected frequency of founders carrying the transgene and had the expected Mendelian distribution of the transgene in multiple founders. However, we did not observe expression or a functional response of PAR1. As a second approach, we targeted human PAR1 with the same promoter (GPIbα-Tg-hPAR1). Once again we observed the expected frequency and distributing of the transgene. Human PAR1 expression was detected in platelets from the GPIbα-Tg-hPAR1 mice by flow cytometry, however, at a lower level than for human platelets. Despite a low level of PAR1 expression, platelets from the GPIbα-Tg-hPAR1 mice did not respond to the PAR1 agonist peptide (SFLLRN). In addition, they did not respond to thrombin when crossed to the PAR4^−/−^ mice. Finally, we used an alternative platelet specific promoter, human α_IIb_, to express human PAR1 (α_IIb_-Tg-hPAR1). Similar to our previous attempts, we obtained the expected number of founders but did not detect PAR1 expression or response in platelets from α_IIb_-Tg-hPAR1 mice. Although unsuccessful, the experiments described in this report provide a resource for future efforts in generating mice expressing PAR1 on their platelets. We provide an experimental framework and offer considerations that will save time and research funds.

## Introduction

Protease activated receptors (PARs) are G-protein-coupled receptors (GPCR) that are activated by proteolytic cleavage of the N-terminus. Human platelets express PAR1 and PAR4 and both contribute to thrombin signaling [1]. In contrast, mouse platelets express PAR3 and PAR4, and thrombin signaling is mediated entirely by PAR4. Although PAR3 does not signal, it facilitates the cleavage of PAR4 at low thrombin concentrations by serving as a cofactor [2], [3]. In addition, PAR3 can modulate the signaling from PAR4 at high agonist concentrations [4].

PAR4 is expressed on the platelets of most species, whereas, PAR1 expression in platelets is species specific. PAR1 is expressed on human, monkey, and guinea pig platelets, but not on canine, rat, murine, and rabbit platelets; these species do express PAR1 in other tissues [5], [6]. The platelets from guinea pig express PAR1, PAR3, and PAR4. The expression of PAR3 complicates the translation to human disease since human platelets do not express PAR3 and, in mice, PAR3 influences PAR4 signaling [4]. Further, some antagonists have species-specific interactions. For example, the PAR1 antagonist vorapaxar does not interact with mouse PAR1 [7]. Mice expressing human proteins would circumvent this potential issue. Finally, there are well-described mouse models of thrombosis in which one could test the specific role of the interaction between PAR1 and PAR4 or PAR1 specific signaling. The PAR profile on platelets from cynomolgus monkeys is comparable to that of human platelets, with the expression of PAR1 and PAR4, and no PAR3 [5]. However, the specific requirements for primate studies make this model impractical for initial preclinical studies. The selective expression of PAR1 has limited the opportunities for assessing the role of a PAR1 antagonist as antithrombotic agents with *in vivo* models.

In the present study we aimed to generate a mouse model that expressed PAR1 in mouse platelets to give us a unique opportunity to examine the individual roles of PAR1 and the PAR1-PAR4 heterodimer in platelet signaling *in vivo* by endogenous agonists. Mice expressing PAR1 on their platelets would also allow investigations into specific signaling pathways by crossing these mice with strains that have genetically altered signaling pathways. These studies would also generate a tool to characterize novel PAR antagonists. We chose a transgenic approach using three separate constructs and two different promoters (GPIbα and α_IIb_) that were ultimately unsuccessful at achieving sufficient PAR1 expression in mouse platelets. In each case we obtained the expected number of genetically positive founders. In this report we detail the transgenic approach that was unsuccessful in generating mice expressing PAR1 on their platelets and offer alternative strategies to generate an extremely valuable tool for the cardiovascular field.

## Materials and Methods

### Ethics statement

All animal studies were approved by the Institutional Animal Care and Use Committee at Case Western Reserve University School of Medicine. Human platelets were used as controls in some experiments. These studies were approved by the Case Western Reserve University Institutional Review Board and written informed consent was obtained from all donors.

### Reagents and antibodies

Human α-thrombin (specific activity of 5380 NIH units/mg) was purchased from Haematological Technologies (Essex Junction, VT). PAR1 activating peptide (SFLLRN-NH2) was synthesized at PolyPeptide Laboratories (San Diego, CA). Fura-2AM was purchased from Invitrogen. Prostaglandin I2 was purchased from Calbiochem. The anti-PAR1-PE (WEDE15-PE) antibody was purchased from Beckman Coulter. The anti-P-selectin-FITC (CD62P-FITC) antibodies and JON/A-PE antibodies were purchased from (Emfret Analytics, Germany).

### Plasmid construction

**Figure 1:**
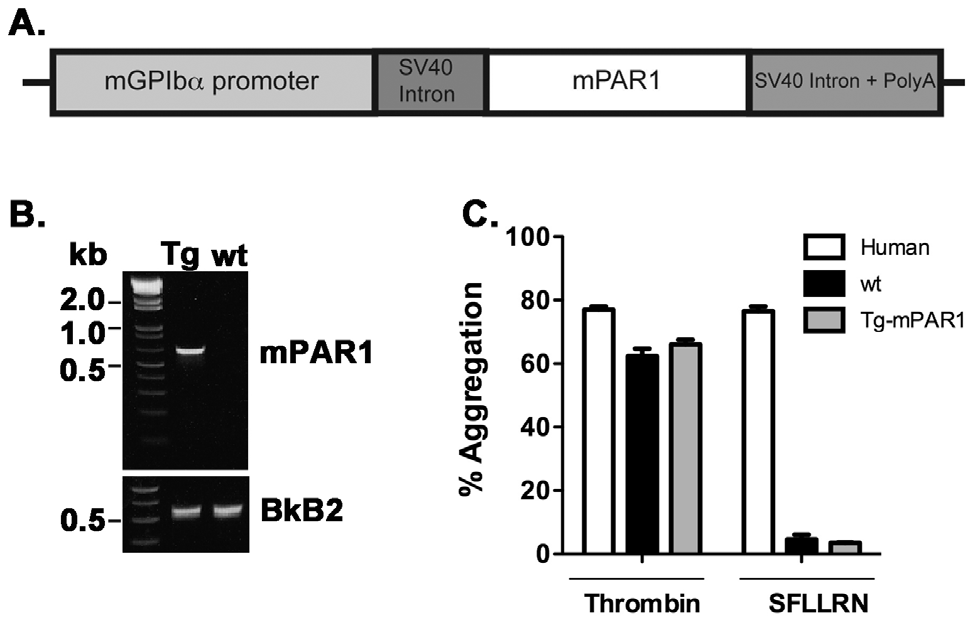
Generation and characterization of transgenic mice expressing mouse PAR1 transgene under control of mouse GPIbα promoter (GPIbα-Tg-mPAR1). (**A**) Schematic representation of the transgene construct. The cDNA for mouse PAR1 was inserted into a vector containing the mouse GPIbα, promoter small-t intron of simian virus 40 (SV40) in the 5′-untranslated region (UTR) and SV40 polyadenylation (polyA) sequence in the 3′ -UTR. (**B**) Representative genotyping from GPIbα-Tg-mPAR1. The control PCR reactions used primers specific for the bradykinin B2 receptor (BkB2). (**C**) Platelet aggregation in response to thrombin (100 nM) or SFLLRN (50 µM) expressed as a percentage of the maximal light transmission. The results are the mean of six independent experiments.

**Figure 2:**
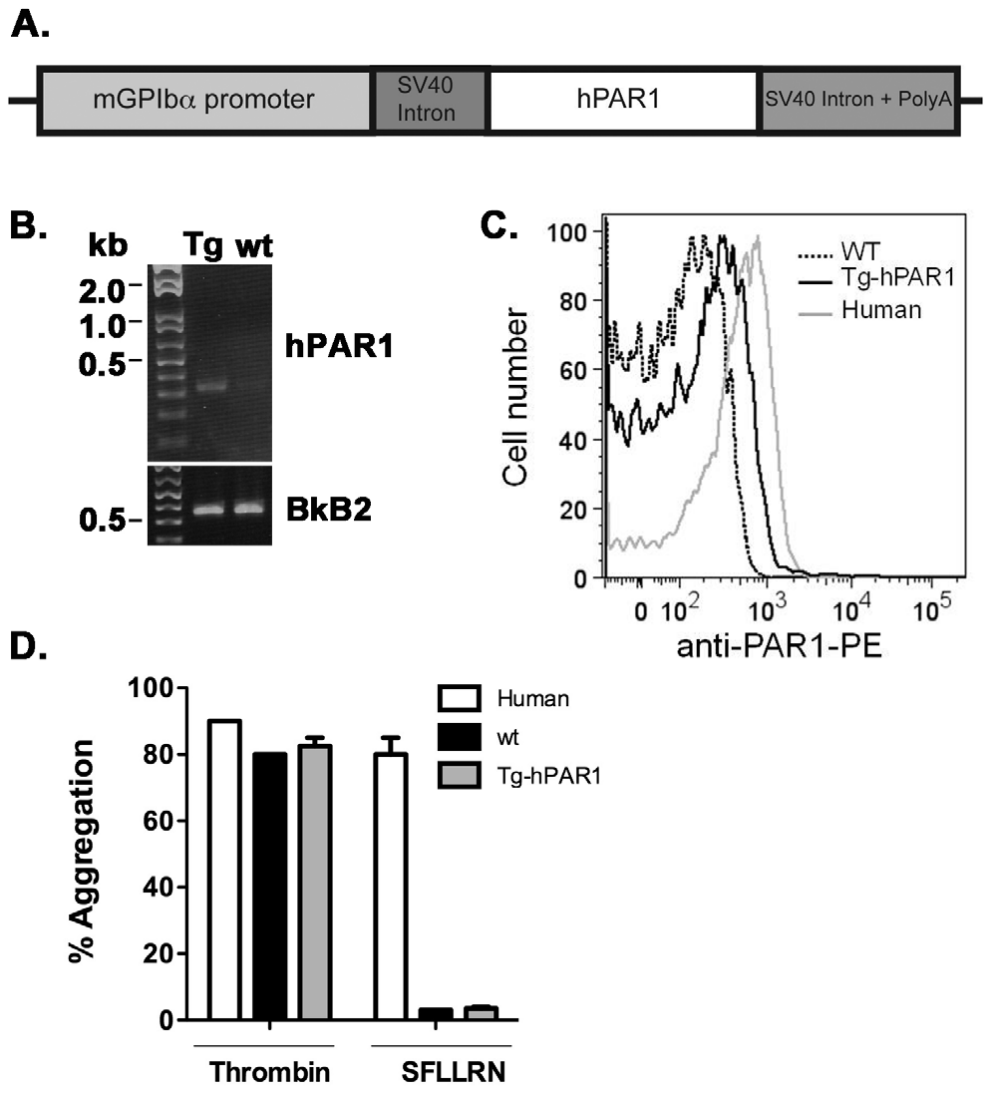
Generation and characterization of transgenic mice expressing human PAR1 transgene under control of mouse GPIbα promoter (GPIbα-Tg-hPAR1). (**A**) Schematic representation of the transgene construct. The coding sequence of the mouse PAR1 was replaced with human PAR1 in the GPIbα vector. (**B**) Representative genotyping from GPIbα-Tg-hPAR1. The control PCR reactions used primers specific for the bradykinin B2 receptor (BkB2). (**C**) Expression of hPAR1 was measured on the surface of platelets from human, wild type mice (wt) and transgenic mice (GPIb-Tg-hPAR1) using anti-PAR1-PE (WEDE15) and analyzed by flow cytometry. (**D**) Platelet aggregation in response to thrombin (100 nM) or SFLLRN (50 µM) expressed as a percentage of the maximal light transmission. The results are the mean of three independent experiments.

The vector containing mouse GPIbα promoter driving Factor VII was obtained from Dr. Mortimer Poncz (Children's Hospital of Philidelphia) [8]. The cDNA for Factor VII was replaced with that of mouse or human PAR1 (see Figure 1 and Figure 2). The human α_IIb_ promoter was kindly provided by Dr. David Wilcox (Medical College of Wisconsin) [9]. The vector for PAR1 expression driven by the α_IIb_ promoter in platelets was generated by replacing the GPIbα promoter. In addition, the Kozak sequence was placed in front of the PAR1 cDNA to enhance expression.

### Animals

Transgenic mice were generated at the Case Western Reserve University Transgenic and Targeting Core Facility. The 4.2 kb transgene (promoter, cDNA, and poly(A)) were released from the vector backbone with a SalI digest, purified, and injected into fertilized eggs. The transgenic animals with mouse PAR1 targeted to platelets under control of the GPIbα promoter were generated on a B6SJL background. The transgenic animals with human PAR1 targeted to platelets under the control of GPIbα or α_IIb_ promoter were generated on a C57BL6/J background. In all cases, founders were bred with C57BL6/J. C57BL6/J mice were purchased from The Jackson Laboratory (Bar Harbor, Maine). PAR3^−/−^ and PAR4^−/−^ mice were obtained from the Mutant Mouse Regional Resource Center (MMRRC) (Chapel Hill, NC). The PAR3^−/−^ PAR4^−/−^ double knockout mice were generated through breeding. All animals were genotyped with PCR analysis using primers specific for each gene. For PAR1 transgenic mice, multiple sets of primers were used to confirm genotyping results. A detailed list of primers used is available upon request.

### Preparation of mouse and human platelets

Mouse platelet isolation was carried out as previously described [4]. Human platelets were obtained from healthy donors. Whole blood was collected into the anticoagulant acid citrate dextrose (ACD) (2.5% sodium citrate, 71.4 mM citric acid, 2% D-glucose) and centrifuged at 250×*g* for 10 min to isolate PRP. One-third volume of ACD and 1 µM prostaglandin I2 (PGI_2_) were added to PRP and the preparation was centrifuged at 750×*g* for 10 min at room temperature. The platelet pellet was washed once in HEPES-Tyrode's buffer (pH 7.4) containing (1/5) volume of ACD and 1 µM PGI_2_. Washed human platelets were counted on a Hemavet 950FS (Drew Scientific Inc, Waterbury, CT, USA) and the final platelet count adjusted with HEPES-Tyrode's buffer.

### Platelet aggregation

Washed platelets were adjusted to a final concentration of 2×10^8^ platelets/mL. Platelets aggregations were analyzed in an optical aggregometer (Bio/Data Corporation) at 37°C under constant stirring at 1200 rpm.

### Measurement of PAR1 expression in mouse and human platelets

Mouse and human platelets were adjusted to final concentration of 40×10^6^/mL in HEPES-Tyrode's buffer (pH 7.4). Platelets (10×10^5^) were incubated with 20 µL anti-PAR1-PE (WEDE15-PE) antibody at room temperature for 20 min. Platelets samples were diluted to (1∶8), acquired on a Beckman Coulter LSRII (Case Comprehensive Cancer Center Flow Core) and the total of 10,000 events were collected for each sample and analyzed with Flowjo software.

### Measurement of the concentration of free intracellular calcium ([Ca^2+^]_i_)

Intracellular calcium mobilization in response to thrombin or PAR1 agonist peptide were measured fluorometrically in Fura-2-labelled washed mouse platelets as described previously [4].

### Measurement of P-selectin expression and integrin αIIbβ3 activation

Washed platelets were adjusted to final concentration of 40×10^6^ platelets/mL in HEPES-Tyrode's buffer (pH 7.4). Twenty microliter aliquots were activated with agonists for 5 min at 37°C and incubated with 0.5 µg/mL anti-P-selectin-FITC (CD62P-FITC) and JON/A-PE antibodies at room temperature for 15 min and analyzed by flow cytometery as described above.

## Results

### Characterization of transgenic mice expressing mouse PAR1 transgene under control of mouse GPIbα promoter (GPIbα-Tg-mPAR1)

Our initial efforts to generate mice expressing PAR1 on platelets used the mouse glycoprotein Ibα (GPIbα) promoter (kindly provided by Dr.Mortimer Poncz, University of Pennsylvania) [8]. The construct used to generate this transgenic mice was represented in the Figure 1A. At the time of our experiments, the gene targeting and transgenic facility at Case Western Reserve University had higher success rates generating transgenic mice on the B6SJL background. Therefore, our experimental strategy was to generate the transgenic mice on the B6SJLF1/J background and backcross the mice to the C57BL6/J, which would also allow us to breed the mice with the PAR3 knockout (PAR3^−/−^) and PAR4 knockout (PAR4^−/−^) mice. We obtained 65 potential transgenic mice, from which we had 15 founder mice (7 males and 8 females) that were identified as positive for the transgene by PCR analysis (Figure 1B). Primers directed to the bradykinin B2 receptors were used as positive control for the PCR reaction. The founders were bred to C57BL6/J. The litters had the expected number of pups with an even distribution of males and females, and the predicted 50% of pups positive for the *mPAR1* gene.

To characterize transgenic mice (GPIbα-Tg-mPAR1) that were positive for the *mPAR1* transgene we examined mPAR1 protein expression and platelet function. We were unable to detect expression of mPAR1 protein in platelets by flow cytometry or Western blot with multiple PAR1 antibodies (data not shown). Since there are examples of transgenic mice in which extremely low protein levels were able to elicit a functional response [10], [11], we tested for PAR1 function by measuring platelet activation in response to thrombin and PAR1 agonist peptide (SFLLRN). Platelets from the GPIbα-Tg-mPAR1 mice did not respond to 50 µM SFLLRN (Figure 1C), whereas the same concentration of SFLLRN induced human platelet aggregation. Platelets from GPIbα-Tg-mPAR1 mice responded to 100 nM thrombin similar to human and wild type mouse platelets (Figure 1C) indicating that they were functional. In addition, SFLLRN did not stimulate P-selectin surface expression or integrin α_IIb_β_3_ activation in platelets from GPIbα-Tg-mPAR1 mice as measured by flow cytometry (data not shown). These data indicate that the mice that were positive for the *mPAR1* transgene did not express mPAR1 protein.

### Characterization of transgenic mice expressing human PAR1 transgene under control of mouse GPIbαpromoter (GPIbα-Tg-hPAR1)

Since we were unsuccessful in generating transgenic mice with mPAR1 expressed on platelets, we altered the transgene to express human PAR1 (hPAR1). The cDNA for mouse PAR1 was replaced with human PAR1 in the mouse GPIbα vector as shown in Figure 2A. Due to improvements in the efficiency of generating transgenic mice on a C57BL6/J background, we chose this approach to eliminate the need for backcrossing. After screening of 16 potential transgenic mice by PCR analysis, we identified 4 founder mice that were positive for the transgene (Figure 2B). The founders were bred to C57BL6/J. As with the previous transgenic animals, the litters had the expected number of pups with an even distribution of males and females, and the predicted 50% of pups positive for the *hPAR1* gene. We determined the surface expression of hPAR1 in platelets from GPIbα-Tg-hPAR1 mice by flow cytometry (Figure 2C). GPIbα-Tg-hPAR1 mice expressed hPAR1 on their platelets; however the expression level of hPAR1 was very low compared to the human platelets. As expected, PAR1 was not detected on platelets from wild type mice. To investigate whether hPAR1 was functional in mouse platelets, we measured platelet aggregation in response to thrombin and SFLLRN (Figure 2D). Platelets from GPIbα-Tg-hPAR1 or wild type mice did not aggregate in response to 50 µM SFLLRN however, they did respond to thrombin indicating the platelets were functional.

**Figure 3:**
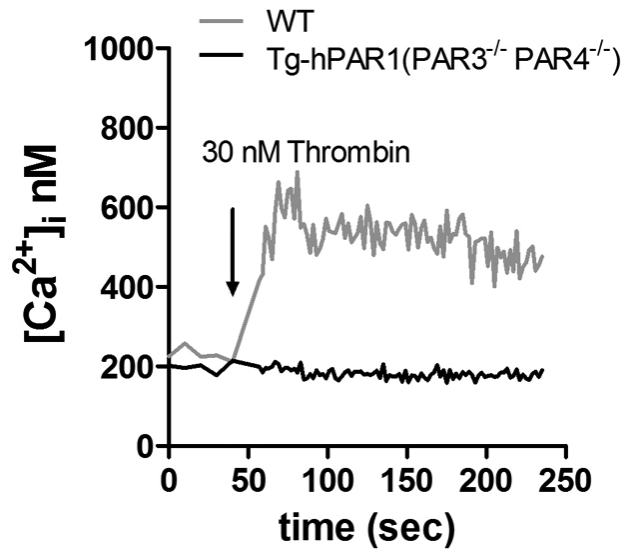
GPIbα-Tg-hPAR1 transgenic mice on a PAR3-PAR4 double knockout (PAR3^−/−^, PAR4^−/−^) background do not respond to thrombin. Intracellular calcium mobilization was measured in platelets from wild type (wt) (gray line) and GPIbα-Tg-hPAR1- PAR3^−/−^ -PAR4^−/−^ (black line) mice in response to thrombin (30 nM). The tracings are representative of three independent experiments.

To be certain that the hPAR1 was not functional in the platelets from the GPIbα-Tg-hPAR1 mice, we wanted to test the response to the endogenous agonist thrombin. To do this without the influence of other PARs, we crossed the GPIbα-Tg-hPAR1 onto the PAR3^−/−^-PAR4^−/−^ background (GPIbα-Tg-hPAR1-PAR3^−/−^-PAR4^−/−^). The PAR3^−/−^-PAR4^−/−^ mice that were identified as positive for the hPAR1 transgene by PCR analysis (data not shown). Calcium mobilization was measured in platelets from GPIbα-Tg-hPAR1-PAR3^−/−^-PAR4^−/−^ mice (Figure 3). In contrast to wild type, Tg-hPAR1-PAR3^−/−^-PAR4^−/−^ platelets did not increase their intracellular calcium in response to 30 nM thrombin. These data confirm that hPAR1 is not functional in the transgenic mice, likely due to low expression.

### Characterization of transgenic mice expressing human PAR1 transgene under control of human α_IIb_ promoter (α_IIb_-Tg-hPAR1)

**Figure 4:**
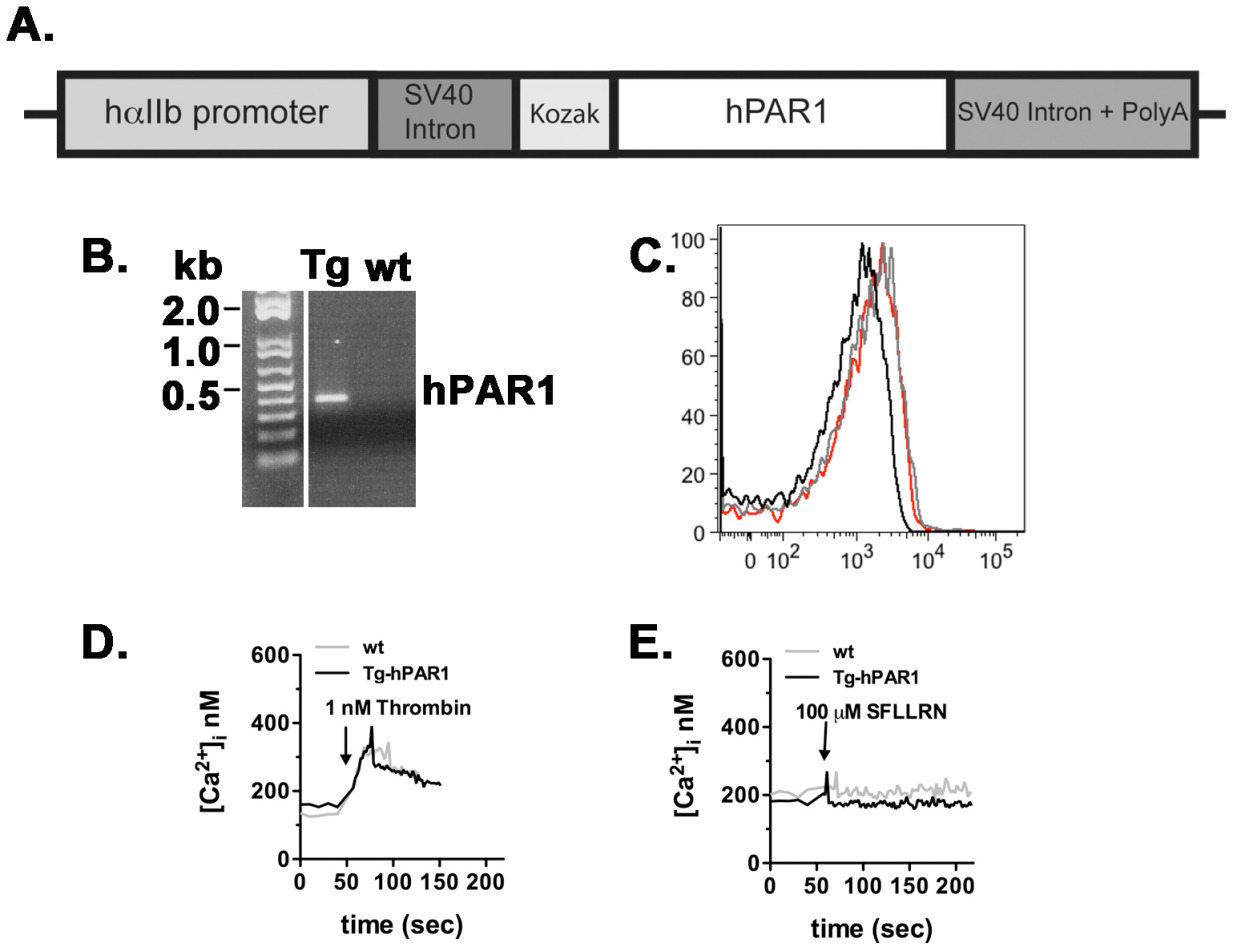
Generation and characterization of transgenic mice (Tg-hPAR1) expressing human PAR1 transgene under control of human α_IIb_ promoter. (**A**) Schematic representation of the transgene construct. The cDNA for human PAR1 was inserted into a vector containing the human α_IIb_ promoter, a small-t intron of simian virus 40 (SV40), and a Kozak sequence at the 5′-untranslated region (UTR) and SV40 polyadenylation (polyA) sequence at the 3′ -UTR. (**B**) Representative genotyping from α_IIb_-Tg-hPAR1. (**C**) Expression of hPAR1 was measured on the surface of platelets from human, wild type mice (wt, black line) and transgenic mice (α_IIb_-Tg-hPAR1) using anti-PAR1-PE (WEDE15, red line) or IgG-PE (gray line) and analyzed by flow cytometry. (**D** and **E**) Intracellular calcium mobilization was measured in platelets from wild type (wt) (gray line) and α_IIb_-Tg-hPAR1 (black line) mice in response to thrombin (1 nM) (**D**) or SFLLRN (100 µM) (**E**). The calcium tracings are representative of three independent experiments.

The GPIbα promoter has been shown to efficiently drive the expression of transgenes in mouse platelets [8]. However, we were unable to detect sufficient expression of either murine or human PAR1 in mouse platelets using GPIbα promoter. Next, we used an alternative platelet-specific promoter human α_IIb_ (kindly provided by Dr. David A Wilcox, Medical College of Wisconsin) [9]. The cDNA for human PAR1 was inserted to the α_IIb_ vector containing a small-t intron of simian virus 40 (SV40), Kozak sequence (GCCGCCACC) at the 5′-untraslated region (UTR), and SV40 polyadenylation (polyA) sequence at the 3′ -UTR (Figure 4A). The Kozak sequence was added upstream of the start codon to increase and improve expression level of hPAR1 in mouse platelets. α_IIb_-Tg-hPAR1 mice were generated on a C57BL6/J background to eliminate the need for backcrossing. We received 19 potential transgenic mice, from which we identified 4 founder mice (2 males and 2 females) that were positive for the transgene by PCR analysis (Figure 4B). As with the previous transgenic animals, the litters had the expected number of pups with an even distribution of males and females and the predicted 50% of pups positive for the *hPAR1 trans*gene. Human PAR1 expression was examined by flow cytometry (Figure 4C). There was no detectable hPAR1 in the genetically positive mice. To fully characterize these mice, functional studies were performed on the platelets from α_IIb_-Tg-hPAR1 mice (Figure 4D and E). In response to 1 nM thrombin the intracellular calcium was increased in platelets from α_IIb_-Tg-hPAR1 mice to the same level as the wild type (Figure 4D). However, there was no increase in the intracellular calcium in response to 100 µM SFLLRN in platelets from α_IIb_-Tg-hPAR1 mice (Figure 4E). As expected wild type platelets did not increase the intracellular calcium in response to the same concentration of SFLLRN (Figure 4E).

**Figure 5:**
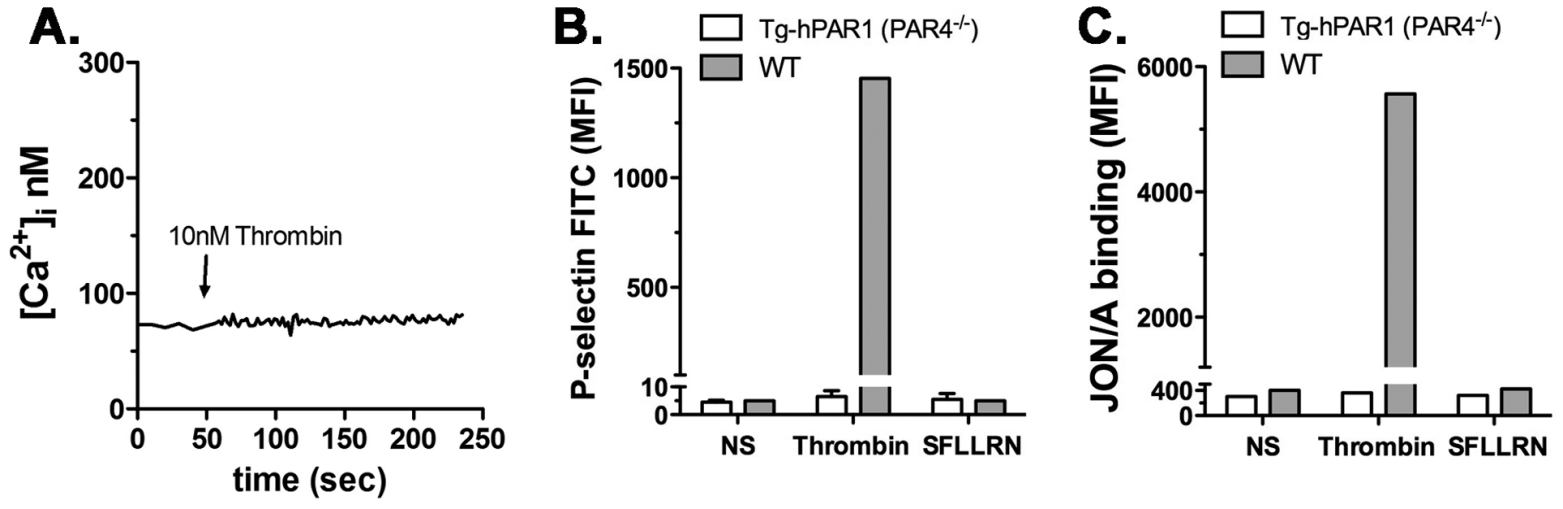
α_IIb_-Tg-hPAR1 transgenic mice on a PAR4^−/−^ background do not respond to thrombin. (**A**) Intracellular calcium mobilization was measured in platelets from α_IIb_-Tg-hPAR1-PAR4^−/−^ mice in response to thrombin (10 nM). The tracing is representative of three independent experiments. (**B**) P-selectin expression was measured in the surface of platelets from wild type (wt) (gray bars) or α_IIb_-Tg-hPAR1-PAR4^−/−^ (white bars) mice by flow cytometry using FITC conjugated P-selectin antibody in response to thrombin (10 nM) or SFLLRN (30 µM). (**C**) Platelets were treated as in (B) and integrin αIIbβ3 activation was measured using PE conjugated JON/A antibody. The results are the mean of three independent experiments.

We were unable to detect the expression of hPAR1 in the transgenic mice platelets using the promoter α_IIb_. We decided to examine the functional response of hPAR1 to thrombin by generating transgenic mice with PAR4 knockout (PAR4^−/−^) background (α_IIb_-Tg-hPAR1-PAR4^−/−^). The α_IIb_-Tg-hPAR1-PAR4^−/−^ mice that were identified as positive for the transgene by PCR analysis (data not shown). Activation of platelets from α_IIb_-Tg-hPAR1-PAR4^−/−^ mice with 10 nM thrombin did not induced intracellular calcium mobilization (Figure 5A). In addition, 10 nM thrombin or 30 µM SFLLRN did not stimulate P-selectin surface expression (Figure 5B) or integrin α_IIb_β_3_ activation (Figure 5C) as measured by flow cytometry. As expected, platelets from wild type responded to thrombin, but not SFLLRN (Figure 5B and C).

## Discussion

It has been well documented that human platelets express PAR1 and PAR4, whereas mouse platelets express PAR3 and PAR4. This species-specific expression profile has limited the ability to use mouse models to examine the individual roles of PAR1 and PAR4 on platelets and preclinical testing of pharmacologic agents. The aim of the current study was to generate a mouse model expressing PAR1 in platelets. These mice would allow us to determine the role of PAR1 in primary hemostasis and platelet thrombus formation *in vivo*. These mice would also allow us to examine the interplay between PAR1 and other platelet receptors in vivo. Although we were unsuccessful at generating mice with sufficient expression of PAR1 on their platelets, our studies provide useful insights and potential alternative methods for future studies generating mice with humanized PAR1 expression on their platelets.

In the current study, we have used mouse glycoprotein Ibα (mGPIbα) promoter to direct the expression of mouse or human PAR1 into mouse platelets (Figure 1A and Figure 2A) or the human α_IIb_ promoter to express human PAR1 (Figure 4A). Although we obtained the expected number of genetically positive animals in each of the transgenic lines, we did not have any mice positive for PAR1 expression or function, except for GPIbα-Tg-hPAR1, where the expression level of PAR1 was detected in mouse platelets but at lower level compared to PAR1 expression in human platelets. Our transgene construct using the α_IIb_ promoter added the Kozak sequence upstream of the start codon in order to optimize hPAR1 translation [12]. This modification did not increase PAR1 expression. There are multiple reasons for having a varied level of expression in transgenic animals. First, the transgene may insert into a locus that is subject to gene silencing. However, we have examined multiple transgenic mice using three separate transgenes making this unlikely. Second, PAR1 may be inherently difficult to express in exogenous systems. In agreement with this, our previous studies have used multiple promoters and constructs in several cell lines and we consistently have lower expression of PAR1 than other GPCRs [4], [13], [14]. In addition, we have difficulties generating stable cell lines expressing PAR1 [14]. A third possibility is that PAR1 expression in platelets may alter cell fate during development [15], [16]. We have bred our transgenic mice to PAR3^−/−^, PAR4^−/−^, or the double knockout (PAR3^−/−^-PAR4^−/−^) and observed the expected litter size and transgenic ratios for each line of animals. Therefore, it is also unlikely that PAR1 is altering cell fate during development. Based on our data, we expect that PAR1 is not stable when exogenously expressed in platelets similar to our observation in cell lines. The primary goal of generating mice expressing PAR1 on their platelets was to develop a research tool to determine specific PAR1 signaling events on platelets *in vivo* in thrombosis models, therefore we did not further explore the precise mechanism for the absence of PAR1 expression in mouse platelets.

The GPIbα and α_IIb_ promoters have been successfully used to generate platelet specific expression of transgenes in previous studies [8], [9]. An alternative strategy would be to use another platelet specific promoter such as the platelet factor 4 (PF4) promoter. However, given our difficulties with expressing PAR1 with multiple platelet specific promoters the PF4 promoter is also unlikely to give sufficient expression. Bacterial artificial chromosome (BAC) transgenic can also offer increased expression of the transgene over traditional transgenic approaches. Attempts to express the factor V (FV) in platelets using the PF4 BAC transgene offered only marginal improvements in expression [10], [11]. There have been successful studies generating PAR1 transgenic animals in other tissues [17], [18]. Therefore, the difficulties in expression may be due to the platelet specific promoters used in this study. One could envision using a global gene such as actin to deliver platelet specific expression. The resulting animals would have PAR1 overexpressed in other tissues, which may have consequences for *in vivo* experiments. More complex strategies have been described to generate inducible expression of platelet specific transgenes and could be applied to PAR1 [19]. Since mice express PAR3, but not PAR1 and human platelet express PAR1 but not PAR3, an elegant approach would be to knock human PAR1 into the PAR3 locus. This approach would have the benefit of simultaneously inserting human PAR1 and deleting mouse PAR3. Recent advances in gene targeting, such as TALEN or CRISPR approaches make this strategy more attracting than in the past. Finally, human PAR1 could be expressed in mouse platelets using a fetal liver cell transplant. With this approach, fetal liver cells are isolated from donor mice and transduced with lentivirus or retrovirus to express hPAR1. The transduced cells are transplanted to irradiated recipient mice. This approach has also been unsuccessful in our hands. The difficulty appears to be in generating cells that stably express PAR1. These data are similar to our cell line experiments described above.

We have discussed alternative methods for generating mice expressing PAR1 on their platelets. There are other considerations that must be taken into account in order for these mice to reflect human disease. For example, we may need to replace mouse PAR4 with human PAR4 in mouse platelets. We have shown in previous work that the calcium signaling is increased in PAR3 knockout mouse platelets expressing mouse PAR4 alone compared to wild type platelets [4]. These data demonstrate that the receptors interact to influence signaling. However, whether PAR3 interact with human PAR4 and influence signaling is not known. Further, during the course of the current study, antagonists to platelet GPCRs have been developed that target the C-terminus of the receptors [20]. The sensitivity of this antagonist was limited to GPCRs that contain a palmitoylation site at the C-terminus of helix 8. In this study, Dowal et al. demonstrated that the antagonist interacts and inhibits mouse PAR4 but not human PAR4. Mouse PAR4 contains a Cys residue at the C-terminal end of helix 8 that is expected to be palmitoylated. In contrast, human PAR4 does not have the Cys residue and contains Gly residues, which can disrupt alpha helices. Given these differences, the best approach may be to completely humanize mice with respect to PARs by also knocking in human PAR4. In this light, the extensive efforts that will be required to generate such animals (double knock-in of hPAR1 and hPAR4) will have to be carefully considered in regards to the potential benefits.

In summary, our extensive efforts to generate mice expressing PAR1 on their platelets using a transgenic approach were unsuccessful. In this report we describe our attempts to express PAR1 in mouse platelets with the hope it will guide others in the field that are interested in generating these mice. Furthermore, we offer alternative strategies and considerations that will be required for interpreting data using a mixture of human and mouse PARs on platelets.
